# Tibial periosteum flap combined with autologous bone grafting in the treatment of Gustilo-IIIB/IIIC open tibial fractures

**DOI:** 10.1515/med-2024-1038

**Published:** 2024-09-20

**Authors:** Yuling Gao, Yang Liu, Hongyu Hu, Shunhong Gao, Junlin Zhou

**Affiliations:** Beijing Chaoyang Hospital, Capital Medical University, Beijing, 100020, China; Department of Hand Surgery, The Second Hospital of Tangshan, Tangshan, Hebei, 063000, China; Department of Hand Surgery, The Second Hospital of Tangshan, Jianshebei Road 21#, Tangshan, Hebei, 063000, China; Beijing Chaoyang Hospital, Capital Medical University, Gongtinan Road 8#, Beijing, 100020, China

**Keywords:** Gustilo-IIIB/III C injury, bone-skin defect, flap, periosteal flap, tibial fracture

## Abstract

**Purpose:**

Gustilo IIIB/C injuries are common for tibia diaphysis fractures with high rates of nonunion, osteomyelitis, and amputation. However, the managements on tibial Gustilo IIIB/C injuries are still controversial and individual. The aim of this study is to introduce the tibial periosteum flap combined with autologous bone grafting to treat Gustilo-IIIB/IIIC injuries.

**Methods:**

Sixteen Gustilo type IIIB/C tibial fracture patients who underwent tibial periosteum flaps with autologous bone grafting surgeries were retrospectively studied. In the first stage, the wound was treated with debridement and the fracture was reduced and fixed with an external fixator. After covering with vacuum sealing drainage for 7 days, the wound areas were repaired by flaps. When the flaps survived and external fixators were removed, the tibial periosteum flaps were taken with autologous bone grafting for bone defects.

**Results:**

The tibia fractures were comminuted fractures with mean size of segment bone defects 3.1 ± 1.3 cm. All the flaps survived and the wound healed in the first stage after an average of 1.5 ± 0.6 months. The mean size of the flap was 13.2 ± 2.8 cm × 7.3 ± 3.1 cm. All the autografts healed in 4.5 ± 0.7 months without infection and malunion. There was no pain in the affected limb. The weight-bearing and walking function were restored.

**Conclusion:**

Tibial periosteum flap combined with autologous bone grafting is effective to treat bone–skin defect of leg with Gustilo-IIIB/IIIC injury.

## 1 Introduction

Open fractures are common for tibia diaphysis fractures which can rate up to 24%, due to its subcutaneous location [1]. Open fractures are classified based on the Gustilo-Anderson Classification. Gustilo IIIB are defined as the presence of significant periosteal stripping and Gustilo IIIC injuries are open fractures with associated arterial injury requiring repair. Both have a high risk of nonunion, because of poor vascularity, loss of soft tissue, infection, and critical-sized bone defects. The incidence of nonunion was 28.9–33%, and osteomyelitis was 9.5–11.1% [2]. The amputation rate for Gustilo IIIB/C injuries can even reach as high as 26.8% [3]. Therefore, Gustilo IIIB/C injuries pose a huge challenge to orthopedists.

However, the managements on tibial Gustilo IIIB/C injuries are still controversial and individual. The basic treatment principles focus on repairing bone defects and performing soft tissue reconstruction [4]. The treatments are usually performed in stages and the basic principles include early debridement, soft tissue cover, and bony reconstruction [5].

Finley first reported in 1978 that the periosteal grafts can produce new function bone without bone grafting [6]. Soldado et al. have successfully developed this surgical technique in pediatric patients and gradually expanded indications and donor sites of periosteal flaps [7–9]. The vascularized tibial periosteal graft (VTPG) has been used in musculoskeletal reconstruction in children [10]. However, studies about the VTPG used in the treatment of tibial Gustilo IIIB/C injuries are few. The aim of this study is to introduce the tibial periosteum flap combined with autologous bone grafting to treat Gustilo-IIIB/IIIC injuries.

## 2 Patients and methods

We retrospectively reviewed the patients’ cases between January 2019 and December 2022 and the inclusion criteria are listed as follows: (1) patients diagnosed with Gustilo type IIIB/C open tibial fractures, (2) patients who underwent two stages of reconstruction with free flap for soft tissue coverage followed by the VTPG for bone reconstruction. We excluded patients according to the following criteria: (1) previous surgery on the affected tibia, (2) tibial bone defects caused by malignancy or other non-trauma diseases, and (3) old fractures. Patients’ demographic information, Gustilo classification, causes of injuries, bone defect size, soft tissue loss, and the time for debridement were recorded.

### 2.1 Surgical technique

The surgical technique was divided into two stages. In the first stage, the patient was placed in a supine position and general anesthesia was induced with a pneumatic tourniquet placed on the proximal part of the affected leg. First, soap and running water were used to wash the skin around the wound. Adequate quantity of lavage fluid was used on the affected limbs. All nonviable or questionably viable skin, subcutaneous tissue, fascia, muscle, periosteum, and bone were excised. After 10 min of lavage of povidone iodine, the wound was washed through saline–hydrogen peroxide–saline procedure. The fracture was reduced under direct vision, restored the length and line of force of the leg, and fixed the distal and proximal tibia with external fixator. Vascular injuries were detected and repaired. Skin defects were covered with flaps after the wound tissue stayed fresh and clean. The wound in the donor area of the flap was sutured directly or grafted and wrapped with pressure by a thigh skin graft.

The second stage began when the flap survived and external fixator was removed. During the second stage, the tibial periosteum flaps from the direct periosteal branch of anterior tibial vessel or intermuscular branch of posterior tibial vessel, were taken with autologous bone grafting for bone–skin defect of lower leg under supine position of patients and general anesthesia condition. The steps were as follows: (a) A longitudinal arc incision on the lateral side of the tibial crest according to the location of bone defects. (b) Identification of the vascular periosteal tibia branch with good quality and thick caliber according to the preoperative examination of color Doppler sonography. (c) Identification of the fracture sites and the location relationships between the fractures and the adjacent tibia periosteal branch. (d) The tibial periosteum flap was taken as a pedicled flap. A tibial periosteal incision should be made to cover at least half of the circumference of the tibial fracture site with around 2.0 cm deep fascia pedicle to protect periosteal branch when sometimes deep fascia pedicle attached to periosteal branch. (e) The periosteal flap was excised for preparation in the range of 7.5 cm × 3.5 cm–10.0 cm × 4.0 cm. (f) Clean the fibrous scar tissue, hardened bone and callus at the fracture end, and open the distal and proximal bone marrow cavity. (g) Internal fixation of the locking plate and screw placed outside when the medial periosteal flap is taken, and inside when the lateral periosteal flap is used to avoid placing plates on the periosteal flap and affecting the blood supply of the periosteal flap. (h) The autogenous iliac bone implanted into the bone defect with a length of 2.5–6.5 cm, with an average of 4.0 cm. (i) Rotation of the periosteal flap with vascular pedicle to cover 1/2 or 2/3 circumference of the tibial fracture. (j) Suture with adjacent soft tissue or periosteum with several needles for the fixation of the tibia periosteal flap.

For the postoperative treatment, routine anti-infection, anti-spasm, anticoagulation, heat preservation, and braking treatment were performed after the first stage operation. Passive flexion and extension exercises of adjacent joints (knee and ankle joint) were performed on the second day after the second stage operation. Weight bearing was prohibited within 6 weeks, and partial weight bearing exercises were performed until complete weight bearing after 6 weeks. The X-ray film was re-examined every month after operation until the fracture line disappeared and there was no local tenderness and longitudinal percussion pain.


**Ethics approval:** The study was conducted in accordance with the Declaration of Helsinki and the guidelines for Good Clinical Practice and was approved by the Ethics Committee of affiliated Beijing Chaoyang Hospital of Capital Medical University and the Second Hospital of Tangshan.

## 3 Results

Sixteen patients who were diagnosed as tibial fractures with Gustilo type IIIB/C injuries and who underwent tibial periosteum flaps with autologous bone grafting surgeries were retrospectively studied. The clinical details of patients can be seen in Table 1. There were 12 males and 4 females. The average age was 38.6 ± 10.0 years old ranging from 19 to 51 years old. The cases of affected part of the tibia included three cases in upper tibia, five cases in middle tibia, and eight cases in lower tibia. The open fractures of tibia were classified into 12 cases of Gustilo IIIB injuries and 4 cases of Gustilo IIIC injuries. The causes of injury included ten cases of traffic accidents, four cases of machine crush injuries, and two cases of fall injuries. The preoperative X-rays showed that all tibia fractures are comminuted fractures with mean size of segment bone defects 3.1 ± 1.3 cm ranging from 1.5 to 4.5 cm and combined with ipsilateral fibula fractures. There was no sign of infection in the wound area. The mean time from injury to the debridement operation was 6.2 ± 0.7 h ranging from 4 to 9 h. After debridement, the mean size of soft tissue loss was approximately 9.3 ± 1.8 cm × (7.3 ± 1.2 cm ranging from 7.5–16.2 to 6.1–8.8 cm.

**Table 1:** Clinical details

Patient	Age (years)/sex	Cause	Location of defect	Gustilo type	Reconstruction	Flap size (cm)	Bone defect length	Periosteum flap/size (cm)	Bone union time (months)	Complication
1	30/M	Fall injury	Middle tibia	IIIB	Anterolateral thigh free flap	12 × 8	2.5	7.5 × 3.5	4	None
2	50/M	Fall injury	Lower tibia	IIIC	Anterolateral thigh free flap	13 × 9	4.0	8 × 3	6	Superficial infection
3	34/F	Traffic injury	Upper tibia	IIIB	Gastrocnemius myocutaneous flap	9 × 8	3.0	10 × 3.5	4	None
4	42/M	Machine crush injury	Middle tibia	IIIB	Posterior tibial artery perforator flap	9 × 7	4.5	10 × 4	4.5	None
5	43/F	Traffic injury	Lower tibia	IIIB	Sural neurovascular flap	12 × 7	3.2	9 × 3	4.5	None
6	29/M	Machine crush injury	Middle tibia	IIIC	Gastrocnemius myocutaneous flap	10 × 6	3.0	8 × 3.5	4.5	None
7	49/M	Traffic injury	Upper tibia	IIIB	Gastrocnemius myocutaneous flap	12 × 7	3.3	8.5 × 3.5	4	None
8	56/M	Traffic injury	Lower tibia	IIIB	Sural neurovascular flap	10 × 8	4.0	8 × 4	5	None
9	32/M	Traffic injury	Middle tibia	IIIC	Anterolateral thigh free flap	9 × 8	1.5	7 × 3	4	None
10	19/M	Machine crush injury	Lower tibia	IIIB	Posterior tibial artery perforator flap	18 × 10	2.3	8 × 3	4	None
11	52/M	Machine crush injury	Lower tibia	IIIB	Anterolateral thigh free flap	12 × 7	3.5	8.5 × 3	4.5	None
12	35/M	Traffic injury	Lower tibia	IIIB	Anterolateral thigh free flap(ALTF)	15 × 8	2.5	8 × 3	4.5	None
13	30/M	Traffic injury	Lower tibia	IIIB	Posterior tibial artery perforator flap	13 × 9	2.5	8 × 3	4	None
14	41/M	Traffic injury	Middle tibia	IIIB	Gastrocnemius myocutaneous flap	12 × 8	3.3	8 × 4	4	None
15	32/F	Traffic injury	Upper tibia	IIIC	Anterolateral thigh free flap	10 × 6	4.0	8.5 × 3.5	5.5	None
16	43/F	Traffic injury	Lower tibia	IIIB	Anterolateral thigh free flap	9 × 7	3.0	8 × 4	4	None

All flaps survived and the wound healed in the first stage. Of the four cases of Gustilo IIIC injuries, there were direct anastomosis of posterior tibial artery in two cases and bridging anastomosis of great saphenous vein in two case. The remaining wounds were covered using vacuum sealing drainage (VSD) for 7 days. After the wound tissue stayed fresh and clean, the skin defects were covered with gastrocnemius myocutaneous flap in four cases, sural neurovascular flap in two cases, posterior tibial artery perforator flap in three cases, and anterolateral thigh free flap in seven cases. The mean size of the flap was 13.2 ± 2.8 cm × 7.3 ± 3.1 cm ranging from 9.0–18.2 to 6.5–10.7 cm.

After an average of 1.5 ± 0.6 months, the second stage was performed. All 16 cases were followed up for an average of 21.5 ± 2.8 months ranging from 12 to 30 months. All the autografts healed, and the mean healing time was 4.5 ± 0.7 months ranging from 4 to 6 months. The formation of callus, recanalization of the medullary cavity, and absence of implant loosening or fracture were observed. The affected limb exhibited no pain, and restoration of weight-bearing and walking function was observed. Furthermore, the knee and ankle joints demonstrated excellent mobility without any signs of infection or malunion. The internal fixation was removed in six patients, on an average 22 months post-operation, with a range of 16–30 months. In the latest follow-up, based on the Johner–Wruh tibial fracture evaluation criteria [11], 14 cases achieved an excellent outcome, while one case was classified as good and another one case as fair. The overall rate of excellent and good outcomes reached 93.8%.

### 3.1 Case example

**Figure 1:**
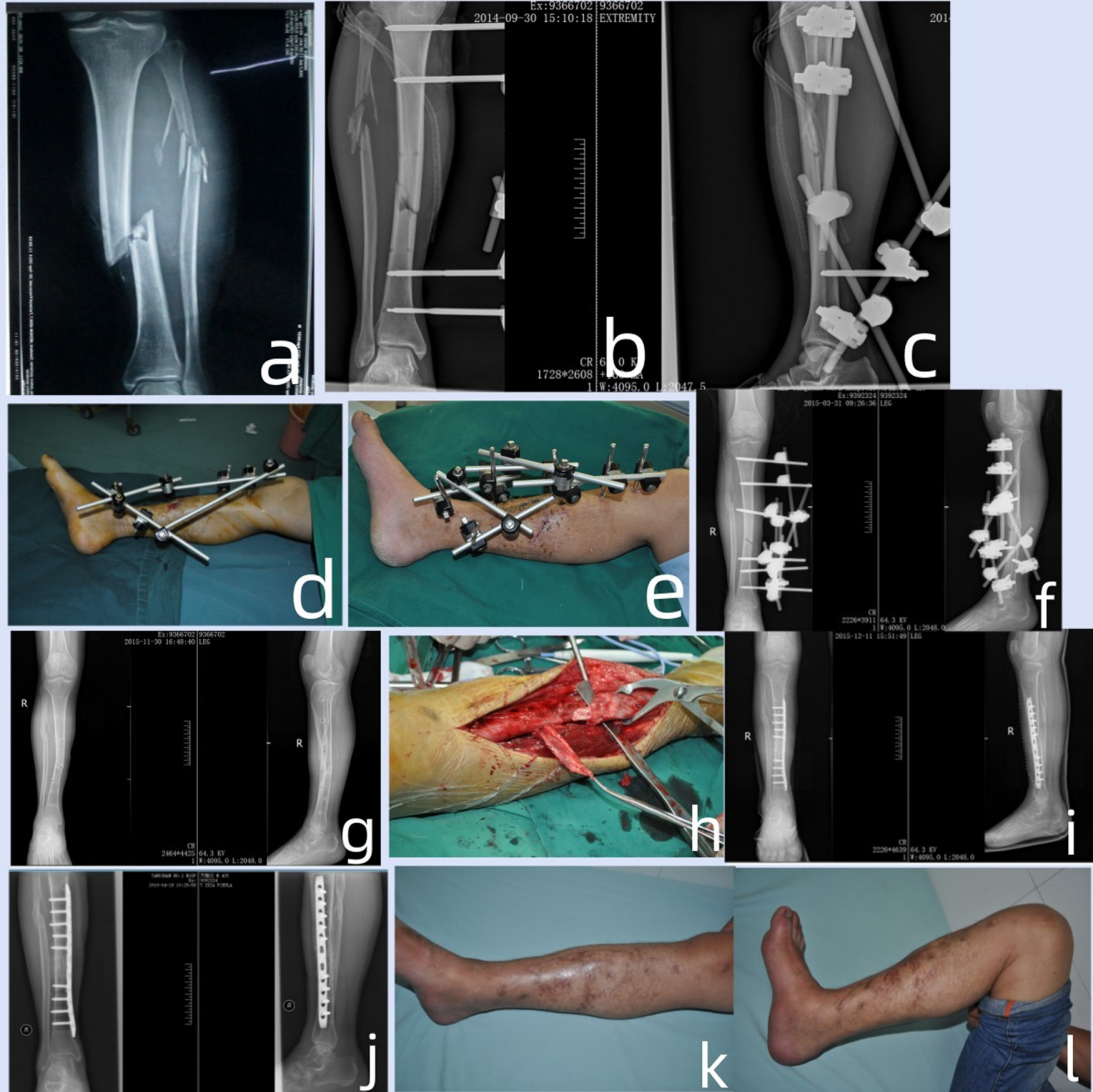
(a) The anteroposterior radiograph showing a Gustilo type IIIB injury with tibial and fibula bone fracture. (b) and (c) The lateral and anteroposterior radiograph after 2 weeks of the external fixator fixation of the affected lower extremity. (d) Appearance of the medial view of the wound after 2 weeks of the external fixator fixation. (e) The wound covered with flap after 2 weeks of the external fixator fixation. (f) and (g) The radiograph of injury after 6 months. (h) The operation of the transposition of the periosteal flap. (i) The radiograph after the internal fixation of tibial fracture with bone graft and periosteal flap transposition. (j) the radiograph showing the bone union after 4 months of the internal fixation of tibial fracture with bone graft and periosteal flap transposition. (k) and (l) The lateral and anterior view after 12 months of injury.

A 19-year-old male suffered a machine injury, resulting in a Gustilo IIIB injury with right distal tibia meta-epiphyseal fracture (AO 43C3). The debridement procedure was performed, maintaining the tibia length through the application of an external fixator, and subsequently covering the wound with a VSD device. After 2 weeks when the wound tissue stayed fresh and clean, the skin flap was repaired with posterior tibial artery perforator flap for 18 × 10 cm size. After 6 months the tibial bone nonunion was detected and the bone defect was 2.3 cm in length. The tibial bone nonunion site was transplanted by the tibial periosteum flap combined with autologous bone grafting and stabilized with a locking plate. The periosteum flap size was 8 × 3 cm. The radiograph showed the bone union 4 months after the internal fixation. The patient was satisfied with the functional outcomes after 12 months after trauma (Figure 1).

### 3.2 Statistical method

The measured data were processed using SPSS 19.0 statistical software. All data were presented as mean ± standard deviation. The operations were performed by one experienced surgeon. The bone defect length and the size of flaps including periosteum flaps were recorded separately by three experienced surgeons.

## 4 Discussion

In this study, we proposed a two-stage treatment protocol for Gustilo IIIB/C open tibial fractures, involving initial flap transplantation followed by subsequent tibial periosteum flap with autologous bone grafting to address the bone–skin defect. Our findings demonstrate the efficacy of VTPG in treating 16 patients diagnosed with Gustilo IIIB/C injuries.

In our study, we took earl repair (≤2 times of debridement) with free flaps on the lower extremities reconstruction. The early-repair group had a less risk of total or partial flap necrosis, lower incidence of complications, and less wound size than the delayed-repair group (>2 times of debridement) [12]. For patients with Gustilo IIIB and IIIC injuries accompanied by significant soft tissue loss, despite successful vascular repair, accurately assessing the viability of the remaining soft tissues remains challenging and necessitates further observation. VSD can obviously promote the growth of granulation tissue and reduce the wound surface area [13]. So all affected legs in our study were fixed with external fixator and covered with VSD. Skin flap translation was performed approximately 1 week later until the wound boundary was clear and the surface was fresh. In the two-stage operative protocols, timing to definitive fixation and flap coverage (2–5 days) was not associated with negative outcomes [14,15]. When selecting the soft tissue coverage, we followed the principle of pedicle flap *priori* over free flap and minimum of the donor site [16]. In this group, nine cases were covered with medial gastrocnemius myocutaneous flap, sural neurovascular flap, and posterior tibial artery perforator flap, which were pedicled flaps without affecting the main vessels flow. For seven cases with the large size of wound and surrounding tissues badly traumatized, the free anterolateral thigh flap was used. The medial sural vessels were anastomosed with the free tissue flaps, and all the flaps survived.

The clinical treatment of bone defect includes non-vascularized bone graft, vascularized bone flap, Ilizarov technique, Masquelet technique, and periosteal flap combined with autogenous bone graft [17]. The traditional treatment are limited by bone size availability, donor site morbidity, lack of osteogenic potential, infection, the burden of circular external fixator, and prolonged treatment time [18]. However, the periosteum has the advantage of minimal ectopic ossification and appropriate vascularization in bone healing [19]. Apart from providing the elastic fibers, collagen, and a vascular network, the periosteum also supports the formation of the chondroblasts and osteoblasts in the cambium layer [20]. Theoretically, any vascularized periosteal flap might be potentially used as a free flap. The VTPG, vascularized fibular periosteal flaps, and free vascularized medial femoral condyle periosteal flaps have been reported in the literature [7,21,22]. The direct perforating branch and communication network of the VTPG are abundant, ensuring ample blood supply. The VTPG can be divided into two periosteal grafts, the posterior one based on intermuscular branch of posterior tibial artery and the anterior one based on direct periosteal branch of anterior tibial artery. The periosteal branches of the anterior artery pass underneath or occasionally through the origin of tibialis anterior. On the periosteum they form rings around the shaft of the tibia with two or three vertically orientated anastomotic channels joining adjacent periosteal rings [23]. It means that a wide range of preferable periosteal flaps, both the medial and lateral periosteum of the tibia, can be selected without affecting the main blood vessels’ supply.

For the surgery techniques, when the lateral tibial periosteal flap is dissected, 1.0–1.5 cm of the tibia periosteum on the anterior edge and medial side of the tibia should be preserved to ensure the integrity of the vascular anastomosis chain at the anterior edge of the tibia and enlarge the VTPG range. We took some tibialis anterior muscle close to it when there is scar tissue around the periosteal flap of the direct perforating branch of the anterior tibial artery so as to ensure the blood supply to the periosteal flap. The tibial periosteal flap of the intermuscular branch of the posterior tibial vessel is located between the soleus and the flexor digitorum longus, where perforating branch is closely related to the deep fascia. So the wide fascial periosteal pedicle of 2.0 cm is preserved as far as possible to ensure the blood supply. When covering the fracture end, the periosteal flap should be sutured and fixed with the surrounding tissue to ensure the full coverage and adhesion of the periosteal flap at the fracture end.

There were several limitations in our study. First, it is not easy to perform a pedicled periosteal flap from a previous surgical treated bone fracture which would increase the difficulty of choosing the perforator vessels of the periosteal flap from the anterior or posterior tibial vessels. Second, the contribution of periosteal flap and bone grafting in bone union need to be further debated.

## 5 Conclusion

Tibial periosteum flap combined with autologous bone grafting is effective to treat bone–skin defect of leg with Gustilo-IIIB/IIIC injury.
